# Mental Wellbeing and Social Resilience of Eritrean Refugees Living in Germany

**DOI:** 10.3390/ijerph191711099

**Published:** 2022-09-05

**Authors:** Temesghen Gebresilassie, Claudia Beiersmann, Sandra Ziegler, Verena Keck, Yonas Semere Kidane, Albrecht Jahn, Janine Benson-Martin

**Affiliations:** 1 Heidelberg Institute of Global Health, Heidelberg University Hospital, 69120 Heidelberg, Germany; 2 Section for Health Equity Studies & Migration, Department of General Practice and Health Services Research, Heidelberg University Hospital, 69120 Heidelberg, Germany; 3 Gesundheitsamt Enzkreis, The Public Health Office Enzkreis, 75177 Pforzheim, Germany

**Keywords:** Eritrean refugees, mental wellbeing, social resilience, Germany, ADAPT model

## Abstract

Mental health and social resilience play a significant role in refugees’ adaptation during the resettlement process in the host country. Maintaining good mental wellbeing helps the refugees to respond to stressful experiences with healthy life choices. This study aimed to explore the mental wellbeing and social resilience of Eritrean refugees living in Germany and to identify social conditions and enablers to foster adaptation. This study employs a qualitative approach with a semi-structured, in-depth interview data collection method. Informants were identified among mostly young adult refugees living in Heidelberg, Germany, with a migration history of 3–6 years. In total, 15 informants were recruited through snowball sampling. Data were sorted and analyzed using the five pillars of the Adaptation and Development after Persecution and Trauma (ADAPT) model. The findings suggest that Eritrean refugees experienced psychological distress after resettlement in Germany; however, with time, their mental health improved. The study revealed conditions that were experienced as hindrances, as well as ones that were considered to be resources of positive mental wellbeing and social resilience for resettled refugees. Resettlement challenges described by the participants were the language barrier, discrimination, unemployment, insecure residence status, loss of family and friends, conflict within the diaspora community, and isolation. The main sources of mental wellbeing and social resilience include the feeling of being welcomed by local communities, access to social services, adopting new relationships, and educational opportunities. These experiences encouraged refugees to have a favorable view of their lives and futures and were also found to facilitate better integration and adaptation. Understanding refugee mental wellbeing and social resilience requires a multidimensional perspective. Eritrean refugees living in Germany have experienced and are still experiencing resettlement challenges, such as, for example, loss of family and friends, negative perception of the German system, loss of past achievements, or unemployment. However, they have developed adaptive and resilience mechanisms, as well, such as seeing an opportunity for a better life, adopting new roles, and accepting Germany as a “second home”. In addressing those issues reported by the refugees as hindrances, these could be turned into sources of mental wellbeing and resilience.

## 1. Introduction

Seeking refuge is one of the oldest practices known to men [1]. People leave their home countries in the face of persecution for reasons of their religious beliefs, ethnic background, or their political and social activities and become refugees [2,3].

In 1951, the United Nations declared an official definition for the Status of Refugees at the United Nations (UN) Geneva Convention, which defined refugees as:

*“A person who, owing to a well-founded fear of being persecuted for reasons of race, religion, nationality, membership of a particular social group or political opinion, is outside the country of his nationality and is unable or, owing to such fear, is unwilling to avail himself of the protection of that country; or is unwilling to return to it”*

[4]

Once refugees reach their destination countries, they often must endure a long, stressful, and uncertain period in detention and asylum centers [5,6]. Lack of information and unfavorable policies coupled with potential hostility towards refugees in the host country all add additional challenges to the refugee’s experiences in the host country [7,8]. These intense experiences will often lead to a high prevalence of mental health distress and disorders among refugees, including depression, anxiety, anger, feelings of isolation, and post-traumatic stress disorder [8,9,10].

Mental wellbeing is not a mere absence of distress and mental disorder; rather, it is a stable and strong state of wellness, satisfaction, and contentment [10]. The WHO describes mental health as “a fundamental element of the resilience, capabilities, and positive adaptation that enable people both to cope with adversity and to reach their full potential and humanity” [11]. Resilience is “the process of negotiating, managing, and adapting to significant sources of stress or trauma” [12]. Mental wellbeing incorporates resilience as an essential part of wellness; for example, individuals’ own ability to cope with stressors, be part of a community, and to be able to learn and socialize are some of the signs of mental wellbeing [13,14,15].

Additionally, studies show improvement of mental wellbeing among refugees is influenced by successful utilization of social resilience [16,17]. Social resilience is defined as an adaptive system that encompasses “individuals’ and societies’ capacity to acquire new strategies for survival and cultural creativity to embrace and adopt new contexts to maintain normal function without fundamental loss of identity” [18,19]. Mental wellbeing and social resilience are interconnected, as the refugees’ ability to react and adapt to adverse and traumatizing experiences is associated with social relationships [19,20].

Refugee mental health and wellbeing have been addressed by previous studies with varying findings. A survey study of 4325 recently resettled adult refugees in Germany showed that the majority of participants show severe symptoms of depression, anxiety, and lower life satisfaction owing to uncertain legal status, family separation, and living in refugee housing [21]. Another recent study among asylum seekers and refugees in Germany also found that the level of mental distress was higher than that of the local population, with 46% and 45% of asylum seekers and refugees suffering from depression and anxiety, respectively [22].

Eritrean refugees make up a large number of refugees living in Germany [23]. In 2018 alone, around 10,200 Eritrean refugees reached Germany and applied for asylum [24]. Even though currently, Eritrea represents one of the highest numbers of refugees from sub-Saharan Africa, it has not actually been at war for almost two decades. Nicknamed “national slavery”, most Eritrean refugees cite indefinite conscription into the national service as the main reason for fleeing the country [25]. The Eritrean national service proclamation was declared in 1994. In the beginning, every eligible citizen between the ages of 18 to 40 was expected to serve for 18 months. However, a campaign by the Eritrean government in 2002 changed the duration period of national service to indefinite [25]. Other reasons mentioned as factors for leaving Eritrea include banning all civil society organizations, lack of freedom of speech, religion and political opinion, human rights abuse, imprisonment, and forced labor [25].

### Models for Social Resilience

Different models of resilience have been developed which focus on the environment, the institutions, and societal resources for promoting refugees’ social resilience and mental wellbeing. One example is the ADAPT model (Adaptation and Development after Persecution and Trauma) [26]. This model helps to understand the experience of refugees in resettlement and the effects it has on their mental health and social organization. According to the ADAPT model, individuals and societies have an innate drive to survive and adapt, but the effectiveness of these natural drives depends on the availability of social resources.

According to Tay and Silove [27], the ADAPT model provides a conceptual framework to understand refugees’ experiences and their collective social responses to the challenges they face. The model projects that by addressing the social dysfunction and resilience capabilities of refugees, their mental wellbeing could also be positively affected.

The authors of the ADAPT model [27] have formulated five key interrelated pillars of strategies or “adaptive systems” which could be affected after experiencing forced displacement and resettlement. According to Silove [26], these five pillars represent a strong base for the development of a stable community and society. The five pillars include security and safety (1), interpersonal bonds and networks (2), access to justice and protection (3), social roles and maintained identities (4), and the availability of institutions that confer existential meaning and coherence (5). The ADAPT model further provides principles for policies and practices for mental wellbeing and social resilience interventions to create a holistic recovery in response to challenges faced by refugees [26].

Generally, little attention is given to refugees’ resettlement challenges in the broad context of the social environment. Hence, the current study used a qualitative design to provide multidimensional information about refugee resettlement experiences; it explores the mental wellbeing and social resilience mechanisms of Eritrean refugees living in Germany. This study aimed to understand the Eritrean refugee’s perceptions of their mental wellbeing, identify the social factors and conditions in Germany that may be associated with mental wellbeing and social resilience, and determine the measures taken by Eritrean refugees to adapt to the social challenges faced in the host country and examine the sources and hindrances for Eritrean refugees’ development of social resilience.

## 2. Materials and Methods

### 2.1. Study Design

The current study adopted a qualitative approach with a phenomenological design, as it was ideal for identifying and studying the experiences and issues from the perspective of the study participants. The phenomenological design focuses on gathering detailed individual information, perceptions, and experiences within a particular group of people to understand and describe a particular phenomenon [28].

### 2.2. Study Setting and Participants

The study involved 11 male and 4 female participants. The number of participants was a result of the accessibility of participants, speed of the snowball method (see Section 2.3), availability of resources (primarily time), and participants’ willingness to participate. The study participants were all recruited within the state of Baden-Württemberg in Germany. Eritrean refugees between the age of 18 and 40 who came to Germany between 3 and 6 years ago were recruited, thus ensuring a source of ample post-migration experience of life in Germany as well as limiting the years spent in Germany that can affect the experience. The rationale for the age selection was that the majority of the refugees who arrive in Germany are under the age of 35 [29].

### 2.3. Sample Technique and Data Collection Tools

The exponential non-discriminative snowball sampling method was the most appropriate for this study as there is no complete sampling frame from which to draw a random sample. In this sampling type, the first participant is recruited and provided multiple referrals; thereafter, the recruited participant in the research work recruits another participant until the needed number is reached [30]. The initial male participants were recruited from a local barbershop owned and managed by Eritreans, as well as from a restaurant that is frequented by local Eritrean residents. The initial female participants were recruited from a local Eritrean orthodox church community.

To explore and understand the details of refugee experiences, the researcher adopted a semi-structured in-depth interview technique with open-ended questions as a data collection method. The interview guide was designed based on a preliminary literature review of the conceptual framework used for the study, i.e., the ADAPT model [26]. Participants’ experiences with a focus on challenges encountered since arrival in the host country were the focus of the interviews, as well as their coping skills and self-identified resources utilized for social resilience. For mental wellbeing, no specific framework was used. Instead, we relied on the self-reflective description from the participants and adapted it to the ADOPT model framework.

The interview guide was reviewed by scholars and professionals who worked in the fields of public health, anthropology, and psychology. It also went through another review by conducting two trial interviews. Based on the recommendations and advice given by the scholars and professionals and the trial interview, the guide was slightly modified to serve as an optimal basis for the communicative exploration of refugee experiences.

### 2.4. Data Collection Procedures

Due to the recent COVID-19 pandemic, face-to-face social interactions were prohibited during the data collection period; thus, all interviews were conducted via telephone. The researcher and all participants were able to speak and understand Tigrinya (the official Eritrean language); therefore, the interview guide was translated from English to Tigrinya by the principal researcher (T.G.) and Eritrean peer reviewer (Y.K.G.), and all the interviews were conducted in Tigrinya. This helped the participants express their ideas, views, and experiences with ease.

Information sheets and consent forms, written in Tigrinya, were mailed to the participants before the actual dates of the interviews. Because there could have been illiterate persons, the information sheet and consent form were read out to the interviewee before the start of the interviews. All participants had given their consent to the interviews being audio recorded, analyzed, and published.

### 2.5. Data Analysis

Thematic analysis was used in the study to interpret and code the data. For the analysis, a systematic and iterative process was utilized. This process consisted of multiple stages: the first stage was the verbatim transcription of the interviews, and the next step was to translate the transcribed data into English. The process of translating Tigrinya to English was completed by the principal researcher with a detailed and careful assessment of each word. Necessary alterations had to be made, as the grammatical structure of Tigrinya (Geez) is different from English [31]. The translated texts were later back-translated into Tigrinya by another Eritrean peer reviewer (Y.K.G.) with advanced skills/proficiency in both languages to ensure the quality of the translated data.

Relevant segments from the translated transcripts were condensed into codes and given the appropriate label by the researcher. NVivo software (QSR International Pty Ltd., Doncaster, Australia (2018), NVivo Version 12) [32] was used for coding and analysis of the data set.

The themes of the study were developed according to the five themes described in the ADAPT framework, which allowed for a unique understanding of the issues being examined here.

### 2.6. Ethical Consideration

The study protocol had been submitted before the start of the research, and ethical approval was obtained from the ethics committee of the medical faculty of Heidelberg University (S-168/2020). The study was conducted under the principles described in the Declaration of Helsinki [33].

## 3. Results

The results of the current study are presented in two sections. The first section consists of the demographic data of the participants. The second section describes the self-reported status of mental wellbeing and subsequently reports on the five pillars of the ADAPT model (safety and security; interpersonal bond; sense of justice; role, identity, cultures; and meaning and coherence). Finally, hindrances and sources of social resilience are presented and summarized.

### 3.1. Demographics of the Participants

A total of 15 participants took part in the study, including 11 males and 4 females. Most of the participants were young males between the ages of 18 and 24. The majority of the participants were either employed or were currently enrolled in language or vocational training. Three participants had a B1 level of German language proficiency, and 11 participants either had no academic language proficiency or only had an A1 level of proficiency. One participant had a C1 level of German language proficiency. More details about the participants are shown in Table 1.

### 3.2. Self-Reported Status of Mental Wellbeing

When discussing their mental wellbeing, almost all the participants described their current mental wellbeing as having improved since arriving in Germany; the first years were characterized by all the participants as “distressing, shocking, and stressful”.

*“In the beginning, it was stressful; we were suffering. My husband and I did not know anything about Germany, so everything was a shock for us.”* (female, 18–24).

Time was mentioned as a healer. With the length of the time spent in Germany, all participants reported their mental wellbeing improved. Most of the participants reported incidents of distress and depression in the first years of the resettlement process, with two of the participants taking medication for the depression they suffered.

Some of the mentally stressful experiences reported by the participants are isolation and feeling of loneliness. The most common source of stress was the “language barrier”; other reasons for stress were the burden of responsibility for family back home, unemployment, and cultural differences. A unique source of stress expressed by more than half of the participants was related to receiving many official letters and contracts through the mail. Specifically, difficulty reading the letters and the quantity of the letters received were reported as the source of stress.

*“One thing I hate about Germany is that they have too much paperwork for little things, and they send you so many letters and that drove me crazy in the early days. I still hate it, and whenever I go to check the mail, I get stressed.”* (female, 18–24)

### 3.3. Safety and Security (ADAPT Pillar 1)

Safety and security were discussed as the first part of the five pillars of the ADAPT model. Most of the participants considered German people as *“law-abiding citizens”* (female, 18–24) and the country of Germany as a *“safe place to live”* (male, 31–35, female, 25–30). The perceived strict rules and regulations of the German society were considered as a positive influence on the safety of the participants, and for all the female participants, physical safety was not considered an issue in Germany, stating this:

*“I am safe here […] If someone does anything to me, he will never get away with it. I feel confident about my safety here.”* (female, 25–30)

Another topic discussed along with safety was emotional and physical security; all participants described one or more reasons to be insecure in their current lives. The most frequently mentioned insecurity was related to the residence permit. Insecure and temporary residence permits were reported as an obstacle to integration and as a source of stress by all participants. Some participants described fear of deportation when their residence permit expires; they expressed an uneasy feeling of uncertainty. Some participants were reminded of their past experiences in Eritrea when thinking about this fear. The participants recounted the insecurity they felt when they did not know when they would be taken by the military police to the national service in their home country, known locally as “gefa”, as similar to the uncertainty of not knowing if they would be allowed to stay or not in Germany.

*“…. Just like back home when we had to flee our homes because of “gefa” and here I had the same experience…”* (male, 18–24)

For most participants, safety and security were interrelated with the prospects of their future. Many participants shared a sense of hope for a good future even though the short residence permit made them feel insecure. They linked their prospects with the current opportunities opening up in Germany. The “new” life in Germany was referred to as an opportunity, a platform for a future they did not expect to have in their former hometowns.

### 3.4. Interpersonal Bond (ADAPT Pillar 2)

Participants expressed their loss of immediate family ties after reaching Europe, mentioning how much they missed their family back home. Family support and relationship were cited as primary needs for almost all the participants. The majority of the participants expressed negative emotions and distress due to separation from family, as one participant described: *“I cannot handle the stress here alone, I miss my home and my family.”* (male, 31–35).

All participants mentioned losing their social connections upon arrival in Germany, but 14 out of the 15 participants reported establishing a new relationship with other Eritrean refugees in Germany soon thereafter. The majority of the participants considered these relationships as positive concerning the benefit of socializing and interacting with Eritrean refugees.

*“I feel happy; I get to speak to my brothers in my own language. I enjoy the conversations we have.”* (male, 18–24)

The interaction between Eritreans was viewed as a therapeutic time and stress reliever; participants reported feeling “happy” and “excited” to meet their Eritrean friends, and one participant even described meeting Eritreans as “a social medicine”, Stating that *“it is better than the medicine you take from doctors”* (male, 25–30). The most commonly reported benefit of maintaining interaction with a fellow Eritrean was “ease of communication”.

Relationships with non-Eritreans were mentioned as seeming impersonal, and most participants described their interaction with their classmates, workmates, and neighbors, who were not Eritreans, as *“distant and limited”* (male, 25–30).

Participants in the study also discussed the relationship with the Eritrean diaspora community, older Eritreans who have been living in Germany for decades. Communication and interaction with this group were described as “broken” or “hostile”; some participants even claimed that “the reason they received short residence permits” was due to people from the diaspora community acting as translators, and they assumed they were *“lying to the immigration officers about Eritrean politics, which affects our asylum claim”* (male, 31–35). Another participant described his interaction with the diaspora community like that:

*“I can honestly say, I do not have a relationship with any diaspora community. Even if they see you in the streets, especially the young newcomers, they insult us. If they genuinely cared about us, they could have talked to us and discussed our problems; they are liars! To be frank, they were supposed to guide the young generation because they know everything about Germany, but instead, they made sure to create a conflict with the new generation.”* (male, 18–24)

### 3.5. Sense of Justice (ADAPT Pillar 3)

Participants expressed perceived unfair treatment due to their social status as refugees and as Africans. These participants spoke of a need for understanding and compassion because *“the Eritrean situation is not the same as (that of) other refugees”* (male, 25–30). Some participants asked for support and empathy from the German communities:

*“We Eritreans are fleeing a dictatorial regime that is killing its youth, and the Germans should know this well. They had their experiences with a dictator in the past; they fled the country back then too.”* (male, 25–30)

Meanwhile, another reported phenomenon exemplifying a sense of justice in comparison to the past experiences of injustice was the treatment they received from members of German society. More than half of the respondents reported having been treated well within the German community. Yet, these participants also mentioned incidents of unfair treatment (discrimination) by some local communities. All the participants in the study reported having experienced racism and discrimination. Self-perceived discrimination was thematized by more than half of the participants. A female participant reported on her interactions with local officials: *“the offices I went to have no respect for me. Either they rudely treated me, or they treated me like I am ignorant.”* (female, 25–30).

Another concern regarded as injustice brought up in the interviews was the immigration and asylum laws in Germany; the participants described them as violating their human rights and rights for asylum. The possibility of Eritrea being labeled as a safe country of origin after the peace agreement of 2018, and the therefore heightened probability of asylum claims from Eritrea being rejected, was reported as a source of great stress.

*“They (the German immigration authority) should consider the Eritreans refugee’s situation again. We did not escape because of war, there was no war, we fled because of the tyrant leader there. I think it even got worse after the peace agreement and people are still coming.”* (male, 18–24)

### 3.6. Role, Identity, and Culture (ADAPT Pillar 4)

Participants aged 30 and above mentioned the significance of family ties as vital for social roles and self-identity. The majority of the participants under the age of 30 described adapting quickly to new social roles in resettlement.

The cultural difference was considered confusing and disruptive to traditional roles and self-identity. As participants explained their experiences and challenges with role shifts, they expressed fear of losing their traditional roles. Employment was mentioned as a source of stabilization of traditional roles among Eritrean refugees, as one participant explained about her husband and other Eritrean men who are unemployed:

*“In our culture, men need to work, and they won’t feel like a man if they don’t have a job. A man who stays at home all day is considered less than a man, and this is a source of stress for them (…) they get depressed, and they start drinking…”* (female, 18–24)

Many participants stressed that they lost their identity when they reached Germany; they described the feeling of emptiness, and they also mentioned the feeling of confusion concerning their future roles. All participants reported losing their past credentials in the migration process.

*“I only had the clothes on my back and a small bag on my hand. I had no educational or vocational papers from home, and there was no way to get it because of the political situation.”* (female, 25–30)

Losing their past accomplishments, be it educational or vocational, was perceived as identity loss and, therefore, as stressful. Most participants described the feeling of anxiety and confusion about their roles in life after resettlement.

Another loss described by the participants was a phenomenon referred to by one participant as *“generation loss”* (male, 31–35). Participants said that cultural and national identities, which were supposed to be passed on from generation to generation, were disrupted. They described the “generation gap” created by the unstable relationship with the older generation and being cut off from their home country as a reason for young Eritreans to feel they have lost or are losing their identity.

### 3.7. Existential Meaning and Coherence (ADAPT Pillar 5)

Participants pulled meaning and coherence out of religious and traditional value systems. They described the realization of being in an unfamiliar environment as a shock that had to be processed. The “world” had to be understood anew, and their own life experience had to be given meaning. Religion helped most of the participants in that process. All the respondents reported attending religious services at some point in time. For approximately half of the participants, religion was mentioned as a source of unity and strength as well as a central part of their social lives.

The second source of identity stabilization for some people is traditional values. Older aged participants regarded them as violated in resettlement. For example, four of the participants described the phenomenon of young Eritreans being sexually active before marriage, loss of respect for older aged individuals, and individualism. They voiced concerns about this kind of cultural transformation of adolescent refugees.

*“The underage refugees who were placed with German families, don’t respect their elders and their culture, which will lead to arguments and fights. They are losing their Eritrean heritage because they are living with German families.”* (male, 30–35)

### 3.8. Social Resilience

More than half of the participant’s responses saw the current social resilience among the Eritrean refugee community in Germany as weak and ineffective.

*“It (social resilience) is virtually non-existent with us here, social resilience depends on helping and assisting each other, and we are not doing that genuinely, we only pretend to care! Especially the diaspora in Germany.”* (female, 18–24)

Social resilience was associated with socializing with fellow Eritreans; more than half of the participants reported difficulties finding the opportunity and appropriate location for such meetings. Some of the difficulties participants mentioned in socializing were housing location; they described being assigned housing in rural areas, which prevented them from meeting other Eritreans. Churches and traditional coffee houses were mentioned as essential sources of socialization for three male and all female participants.

Although all participants liked to socialize with fellow Eritrean refugees, the current political division among Eritreans was mentioned as counter-beneficial to the social resilience of Eritrean refugees. Similarly, most of the participants reported social media as having a negative consequence rather than being a means to facilitate social resilience. They described social media’s role as divisive and inciting violence amongst Eritrean refugees, providing a platform for hate speech and bullying.

### 3.9. Sources and Hindrances for the Development of Social Resilience

**Figure 1:**
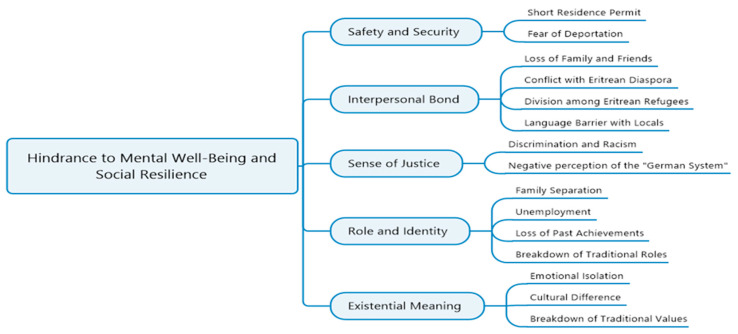
Hindrance factors to mental wellbeing and social resilience (based on the ADAPT model [26]).

**Figure 2:**
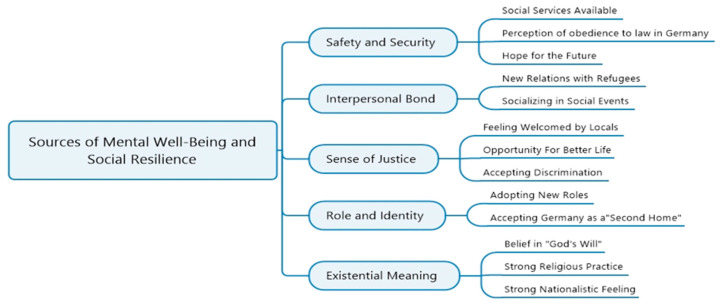
Sources to mental wellbeing and social resilience (based on the ADAPT model [26]).

In line with the results displayed according to the ADAPT model, we identified hindrances and sources of mental wellbeing and social resilience. Hindrances have been attributed to the challenges those participants of this study faced throughout their resettlement process, concluding in negative outcomes for their mental as well as their social wellbeing. Furthermore, our study participants mentioned multiple sources for mental wellbeing and social resilience, which facilitated a positively shaped attitude toward their situation and solidified hope for their future. We visualize these hindrances and sources of social resilience in Figure 1 (hindrances) and Figure 2 (sources).

## 4. Discussion

The result of the study suggests that the experiences of refugees after resettlement are multidimensional and complex. The findings of our study support and contradict previous studies. The post-migration experience of participants was characterized by difficulties, adversities, and discrimination in resettlement but also innovations in integration, utilization of social support, and resources for social resilience.

### 4.1. Mental Wellbeing

All participants gave accounts of their self-reflective psychological experiences and challenges. They repeatedly mentioned experiencing some degree of stress and psychological distress after resettlement in Germany, echoing the finding from a previous study by Biddle [22] on recently resettled refugees in Germany. The current study revealed some conditions which were a hindrance to positive mental wellbeing for resettled refugees. Participants repeatedly mentioned the language barrier as the most common source of stress, which acted as a barrier to their integration into German society. Language difficulties were mentioned in various degrees, resulting in fewer employment opportunities, social interaction, and independence. Similarly, a study by Walther et al. [34] on refugees who resettled in Germany between 2013 and 2016 found that proficiency in the German language was associated with improved mental health. A unique finding on a source of stress in the current study is that of official documentation and letters. Some participants cited the frequency and number of letters received from local authorities, combined with the language barrier to reading the letters, as a factor for post-resettlement stress. Participants explicitly mentioned anxiety due to the accumulation of letters, missing important messages due to a lack of complete understanding of the written information, getting fined because they did not respond to letters sent to them from local authorities and constantly asking people to translate for them. The feeling of being “an outsider” is amplified for refugees with every challenging experience of the language barrier and integration [21].

As mentioned by previous studies [7,9] feelings of isolation are found to be a risk factor for mental health disorders among refugee populations. These feelings are linked to depression, sleep deprivation, thoughts of self-harm, addictions, and risky behavior [9]. By focusing on the challenges of resettlement, the current study findings were able to explore and describe the subjective experiences of post-migration stressors and the influence it has on the mental wellbeing and social resilience of Eritrean refugees.

In general, the level of stress has been reported to decrease with more time spent in Germany. This can be due to multiple reasons, as the participants of the current study are mostly young adults, and so the ability of an individual to recover at this period of their life might be higher compared with older aged individuals [21]. The social environment in which Eritrean refugees find themselves influences their mental recovery as well, and acceptance and social support from local communities help to mitigate past trauma. Additionally, the challenging history of the participants and the previous experiences of migration trauma have contributed to feeling better after resettlement and being grateful for the current experiences. The feelings of hope and optimism appear to be enforced by the knowledge that things could be much worse than they currently are. Similarly, a study by McGregor [35] concludes that previous hardships faced by refugees are mitigated through feelings of appreciation for their current life circumstances.

Mental wellbeing improvement was more evident in female participants, who expressed their overall satisfaction with life, confirming a report by Walther [21] on the refugee population in Germany, which stated that female refugees reported higher life satisfaction compared with male refugees.

### 4.2. Safety and Security

All discussions with the participants addressed the five categories of the ADAPT model in relation to social resilience to varying degrees. Various mechanisms of resilience and recovery, as well as challenges, are discussed.

What facilitated the feeling of safety among participants in the study differed, but the findings show an increased sense of safety. Most participants compared Germany with Eritrea and other countries they encountered during the migration process, which increased the feeling of safety in the current host country.

The general feeling of safety and security was decreased in relation to rights to remain amongst participants as a result of the uncertain and short residence permits they received, which made them question their future, some describing it as “being left in a loop”. Some participants even went as far as comparing their experiences with short residence permits and the fear of deportation with a practice called “gefa” in Eritrea, which is essentially government rounding up of youth for national military service; it is known for being one of the traumatizing experiences of pre-migration for Eritrean refugees [36]. Additionally, some participants described the recent peace agreement between Eritrea and Ethiopia as a source of potential insecurity regarding their residence status in Germany. On 9 July 2018, leaders of Eritrea–Ethiopia met for a peace summit, formally ending the border conflict between both countries, which started in 1998 and continued until 2018 in a frozen conflict state of “no war, no peace” [37]. Short residence status can create a feeling of uncertainty, which hinders Eritrean refugees’ integration process in Germany regarding employment, or language proficiency, confirming the conclusion of a study by Bakker et al. [38] on the adverse effects of insecure residence permits on integration and psychological wellbeing of refugees. Additionally, a lengthy unsecured residence in host countries will “foster a passive attitude towards their future” [38], creating an atmosphere that is not conducive to smooth integration and social resilience.

### 4.3. Interpersonal Bond

Regarding the participant’s interpersonal relationship, the primary source of social interaction indicated by the participants was a relationship created with other Eritrean refugees who arrived in Germany recently, characterizing the relationship as close, full of mutual understanding, and a stress reliever. Social interaction with fellow refugees increases the sense of security and stability of individual refugees and the community as a whole and results in improved reports of mental wellbeing [39]. The positive aspect of socializing with Eritrean refugees was mostly referred to in sentimental tones that centered around nostalgic feelings of home, mother language, culture, and mutual understanding. However, the loss of close family and friends in the process of migration and resettlement was attributed as a stressor among the present study participants. Studies on the mental health and wellbeing of refugees have found that social connections and affiliations are important predictors of successful integration and foster social resilience [2,9]. In addition to the already lost relationships, the interaction with the local residents and Eritrean diaspora communities, who have been living in Germany for decades, did not materialize into meaningful relationships for our participants, making adaptation to the new society an uphill climb. The relations and interactions with the Eritrean diaspora were characterized as hostile, resentful, and suspicious. According to Hepner [40], due to the generational gap, combined with the politicizing of interactions and general miscommunication, the relationship between the newcomer Eritrean refugees and the Eritrean diaspora community has been broken. This phenomenon acted as a hindrance to the utilization of social resources and the development of social resilience. This is not to suggest that all refugees in the current study are unable to access the social resources available to them, but rather, they cannot access the optimal potential of social resources in relation to fellow ex-pats, which would make a positive difference in their integration and social resilience.

### 4.4. Sense of Justice

A sense of injustice before and after resettlement was repeatedly mentioned in the current study. The pre-migration injustice mainly centered around treatment by the Eritrean government, but wrongs and mistreatment committed by the Eritrean government have been reported by some participants as restored by the treatment they received after arriving in Germany. A feeling of injustice in the light of discrimination and racism is also mentioned in previous studies [10,35]. Similarly, in a follow-up study of Syrian refugees in Germany, discrimination was identified as a major risk factor for mental wellbeing [41]. These negative experiences reinforced the challenges of social resilience and adaptation to the new environment, creating a sense of being unwelcome. According to Silove [26], understanding and mitigating the sources of injustice is vital to creating an environment that enhances the process of mental health recovery during the resettlement of refugees.

Refugees with short residence permits typically feel insecure about their future in the host country [5,9]. The participants in the present study echoed a similar sentiment, describing the immigration laws of Germany as unjust since it disregards their unique situation that needed to be addressed differently than that of other refugees, stating they do not see a future where they can go back to their home country anytime soon. It should be noted that this claim has been challenged by the Eritrean diaspora community in a previous study, stating that Eritrea is a safe country [40].

### 4.5. Role and Identity

The findings in this study suggest a link between social role breakdown and the negative mental wellbeing and social resilience of refugees. Our participants pointed to a correlation between social roles (which include employment, traditional gender roles, family roles, and previous social status) and the level of stress expressed by the participants. In line with Sin’s findings [42], the current study especially confirms that employment plays a significant role in social integration and resilience. Unemployment was seen as one of the primary role and identity breakdowns the male participants experienced during their resettlement process. To all female participants of the study, family separation was the most important source of psychological disturbance and challenged their role in the community.

The hostility of the authoritarian Eritrean government towards the refugees who have fled the country makes it impossible to obtain the legal papers needed to confirm past educational and professional achievements [40]. This was seen as an obstacle to resuming their previous vocational role after resettlement. The difficulties in acquiring documentation included their civil documentation as well, mainly marriage and birth certificates, which made it very difficult to reunite with family members. According to Silove [26], oppressive regimes regularly deny individuals social rights to punish dissidents and challenge their sense of identity and control.

### 4.6. Existential Meaning

Religious beliefs play a key role in strengthening the social resilience of refugees; Silove [26] argues that religion solidifies the moral values and beliefs that are integral to social and mental wellbeing. The refugees in the current study reported gratitude and appreciation to God for surviving challenging migration routes and the opportunity to live and pursue their future. The role that religion plays in the social activity of the participants was apparent, especially among the female participants. Strong beliefs and religion have been noted to play a focal role in social interaction and adopting coping mechanisms for refugees resettled in a new environment [20]. A recent study on Eritrean refugees echoed similar findings on the positive role of religious faith, family support, and a vision for the future, which act as enabling factors to build resilience and social interaction [43].

Changes to traditional culture, beliefs, and values took place for the Eritrean refugees while trying to negotiate their new life in the new environment. The new lifestyle encountered by the refugees, which was constantly referred to as “the German system” by the participants of the current study, left them with cultural shock and forced them to innovate and adapt to the new environment. All participants recalled cultural shock when first arriving in Germany, but slowly they grew accustomed to living in their new home. The social and cultural adaptation has not progressed equally for every participant in the study, with more than half of the participants describing difficulties with the “German system” daily. Magnet de Saissy [14] argues rapid and forced adaptation of communities to the new environment will have an impact on the social and mental wellbeing of the individual.

A source of social conflict that stood out in the present study is social media. Participants explicitly mentioned social media tools as facilitators of conflict and division for Eritrean refugees, thus acting as a hindrance to fostering social resilience among Eritrean refugees. This finding contradicted research that found social media as facilitators of positive social interaction for Eritrean and other refugees [19,35]. For most participants of the current study, leaving aside the positive aspect of communication, social media caused harm to the solidarity and social resilience of Eritrean refugees. Some consider the platforms to be a means for the political and regional division to spread among the newcomers. Therefore, this study revealed the negative role social media play among Eritrean refugees.

### 4.7. Social Resilience

The findings of this study demonstrated the importance of a robust social community in building and adopting a resilience mechanism. Identifying and tackling the sources of conflicts among the refugees will be essential to fostering better social interaction and mental wellbeing. Additionally, the study attests to the relevance of the ADAPT model [26] in understanding the complexity and challenging experiences of refugees after resettlement.

### 4.8. Strength and Limitations of the Study

Owed to restrictions due to the COVID-19 pandemic, the study data collection method was limited to a telephone interview; however, we found that this method gave a heightened sense of anonymity to the participants and freedom to speak freely and openly. Additionally, telephone interviews are hypothesized to have a decreasing effect on the social desirability bias [44]. The telephone interviews provided and encouraged an environment of open and honest discussion about culturally sensitive topics, including one’s mental health. However, the absence of visual cues and communication in telephone interviews is a drawback to the natural and emotional interaction in face-to-face interviews and thus produces more restricted responses [44].

The current study was conducted in the frame of a Master’s course (MSc in International Health), with the principal researcher being a young Master’s student of the same national origin as the study participants. Coming from the same socio-cultural background, having a family history of migration as well (although at a very young age), and being able to speak the study participants’ language, Tigrinya, eased access to and familiarization with the study participants as well as building trust to narrate their stories and express their feelings. However, the negative impacts of the researchers’ background on the study cannot be ruled out (e.g., with regards to data collection and analysis).

Furthermore, some of the limitations of the study are the small sample size and the heterogeneity of the snowball sample method. With regards to the small sample size: the fact that the primary researcher had the same socio-cultural background as the study participants might have eased the initiation of the snowball sampling. Yet establishing contact and access to the sampling population, as well as creating trust, takes time. How much COVID-19 also played a role is difficult to say. However, the snowball process went rather slow, and the study had to be conducted within a certain time period (to fulfill the requirements of the MSc). In the end, a total of 15 participants were included.

The time constraints did not allow for planning the study a priori to reach theoretical saturation (as we would not know beforehand how many potential participants contacted would agree to be interviewed, and how many interviews and hence time we would need for this). However, the data were rich enough to answer the study aim, which is one aspect of saturation. Related to that, in qualitative research, numbers (i.e., large sample size) are not as important as the depth of the data [28].

With regards to heterogeneity, 11 male participants and 4 female participants were included. This might present a selection bias, potentially affecting the transferability of our results. Furthermore, inherent in snowball sampling is the fact that the subsequently recruited respondents are more likely to be similar to the previous participants, which could have led to a very concise data picture.

Additionally, female participants being interviewed by a male interviewer could have led to a reporting bias. Sex differences can create a significant barrier to acquiring honest responses to sensitive questions [45].

In qualitative research, the researcher is seen as part of the research process, and it is acknowledged that the researcher comes with their own background, ideas, and interpretations [32]. The researcher should hence always reflect on his/her background and the ways in which this influences the analysis of the data and interpretation of findings. Although efforts were made to stay true to the participant’s response, considering the researcher as only a narrator, and to keep one’s own interpretations apart, it cannot be ruled out that a bias in this direction occurred. In this process, going back to the respondents to dig deeper into the meaning of some data and to clarify the respondents’ view versus the researcher’s interpretation might be necessary. Resource and time constraints, unfortunately, did not allow this in the current study.

Despite these, the current study provides a comprehensive understanding of the current challenges for refugees to feel well and identifies hindrances and sources for better social resilience.

## 5. Conclusions

The findings of the current study can be helpful for the planning of effective and sustainable interventions. One of the ways to mitigate the challenges of refugee resettlement is by focusing on integration lessons and training on topics such as cultures and social and legal systems in Germany, referred to as the “German system” by the participants. The Federal Office for Migration and Refugees (BAMF) provides an integration course on various challenges refugees encounter, consisting of language courses, orientation to administrative regulations, writing emails and letters, and job interviews. The integration course should be tailored to be more culturally appropriate, putting into consideration the understanding and background of refugees. Integration course instructors should be aware of possibly different habits of thought and evaluation as well as daily life practices and experiences of their students, which will help to design the best method to deliver the courses appropriately. Additionally, due to the limited literacy possessed by Eritrean refugees, we recommend German language lessons be already provided alongside the integration courses, preferably instructors/assistant instructors with knowledge of the Tigrinya language; henceforward, they will be able to translate German words directly into Tigrinya words.

Additionally, taking into consideration the lack of access to the proper credentials and documentation, the mentioned loss of past achievements by the respondents could be tackled if government services would be more open to accepting international study and working degrees and vocational training certificates or at least propose flexible ways to catch up with the requirements of the German system. Coupled with efforts to integrate the refugees into job positions, this could not only help to solve Germany’s skilled labor shortage (“Fachkräftemangel”) but also touch upon the mentioned feeling of isolation, as the refugees would be integrated into the “German system”. Easing family identification requirements for the family reunification process could also ease the stress of isolation. By focusing on the challenges of resettlement, the current study findings were able to explore and describe the subjective experiences of post-migrations stressors and the influence it has on the mental wellbeing and social resilience of Eritrean refugees.

In addressing the challenging experiences of refugee resettlement within the five pillars of the ADAPT model, the current study reveals those experiences could be turned into sources of mental wellbeing and resilience.

## Figures and Tables

**Table 1:** Demographic characteristics of participants.

	N
Age range (Years)	
18–24	8
25–30	4
31–35	2
36–40	1
Gender	
Male	11
Female	4
Occupational status	
Married	3
Unmarried	12
Employment Status	
Employed	6
Unemployed	4
Student	5
Stay in Germany (Years)	
3–4	6
4–5	5
5–6	4
German language proficiency	
Low	7
Medium	5
High	3

## Data Availability

The data presented in this study are available on request from the corresponding author.
